# Electro-Mechanically Tunable, Waveguide-Coupled Photonic-Crystal Cavities with Embedded Quantum Dots

**DOI:** 10.1021/acsphotonics.5c00606

**Published:** 2025-07-17

**Authors:** L. A. F. Brunswick, L. Hallacy, R. Dost, E. Clarke, M. S. Skolnick, L. R. Wilson

**Affiliations:** † School of Mathematical and Physical Sciences, University of Sheffield, Sheffield S3 7RH, U.K.; ‡ School of Electrical and Electronic Engineering, University of Sheffield, Sheffield S3 7HQ, U.K.

**Keywords:** nanophotonics, Purcell effect, photonic resonators, microelectromechanical systems, quantum optics

## Abstract

On-chip microcavities with embedded quantum emitters provide an excellent platform for high-performance quantum technologies. A major difficulty for such devices is overcoming the detrimental effects of fluctuations in the device dimensions caused by the limitations of the fabrication processes. We present a system based on a 1D photonic-crystal cavity with an embedded quantum dot. A microelectromechanical cantilever is used to tune the cavity mode wavelength via index modulation and the quantum-confined Stark effect is used to tune the quantum dot emission energy, thus mitigating the effect of fabrication imperfections. To demonstrate the operation of the device, a maximum voltage-controllable cavity tuning range of Δλ = 1.8 nm is observed. This signal is measured at the end of a bus waveguide which side-couples to the cavity, enabling the coupling of multiple cavities to a common waveguide, a key requirement for scale-up in these systems. Additionally, a quantum dot is tuned into resonance with the cavity mode, exhibiting an enhanced emission rate with a detector-resolution limited Purcell factor of *F*_P_ = 3.5.

## Graphical abstract

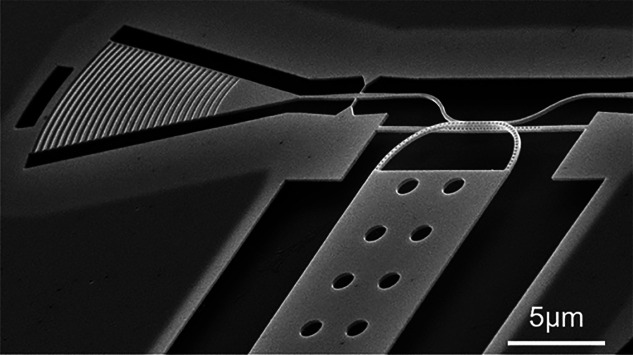

## Introduction

Semiconductor quantum dots (QDs) embedded in nanostructures have been an active field of research for the past two decades, during which significant progress has been made toward the realization of quantum technologies based on this architecture. Demonstrations of key properties such as transform-limited emitter linewidths,^1,2^ highly indistinguishable single-photon emission,^3,4^ strong light-matter interactions^5–9^ and highly efficient, deterministic single-photon generation^10,11^ have established QDs as a leading single-photon source for quantum technology applications.

Embedding QDs in optical resonators such as microcavities enhances many of their favorable properties due to the enhanced light-matter interaction that arises from coupling between the QD and the cavity when they are in resonance. In the weak-coupling regime, this enhancement is known as the Purcell effect^12^ which manifests itself as a reduction in the radiative lifetime of the QD.^13^ This phenomenon not only increases the emission rate of single-photons from the QD, but also reduces the sensitivity to losses caused by decoherence or nonradiative recombination,^14^ which is critical to achieve high performance devices based on single quantum emitters.

To enhance the light-matter interactions of a QD in a microcavity, the QD emission must be in resonance with the optical mode of the cavity. This requirement poses a significant challenge for semiconductor QD systems as, due to the random nature of QD growth and fluctuations in fabrication,^15,16^ it is unlikely that a QD that is located in a cavity will be on resonance with the mode of the cavity. To grow QDs with high-quality optical characteristics the Stranski–Krastanov method is often used, which nucleates QDs in random positions on the substrate, and of random sizes, resulting in a distribution of emission energies.^17^ Furthermore, fabrication imperfections can cause nanostructure dimensions to deviate from their design, leading to significant detuning of the optical modes from their intended wavelength. Consequently, a method to control both the QD emission energy and the cavity resonance is highly desirable to maximize the yield of these devices.

The tuning of QD emission energies is a well-established technique in the field and can be achieved through several different, complementary methods such as electrical tuning via the quantum-confined Stark effect,^18^ strain tuning,^19–22^ magnetic field tuning^23^ and temperature tuning.^24^ When considering cavity tuning methods, they can be split broadly into two categories: material or mode perturbation. Material perturbation methods influence the whole cavity/emitter system by altering the physical properties of the structure. Examples of material perturbation tuning methods include thermal,^25,26^ electro-optical,^27^ acoustic^28^ and strain.^29,30^ Mode perturbation tuning methods modulate the effective refractive index of the cavity mode by using an electromechanical device to control the displacement of a dielectric material within the evanescent field of the cavity mode. This approach has the advantage of preserving the optical properties of the QDs while enabling control of the cavity mode wavelength. Moreover, such an interaction is inherently local to a single cavity, a key requirement for a scaleable cavity emitter system. Such devices have seen success in silicon,^31–37^ diamond - AlN hybrid^38^ and, more recently, in GaAs^39–42^ systems. Actuation can occur in either in-plane or out-of-plane geometries. In-plane actuation requires intricate comb drives, which are complex to fabricate, occupy a large footprint and typically require moderate voltages on the order of 10 V to operate.^43^ Out-of-plane systems of the type we adopt, on the other hand, rely on the electrostatic attraction between layers of the wafer to displace the material, simplifying fabrication and reducing device size.

In the present work, we embed InAs QDs in a 1D-photonic-crystal cavity (PhCC) where the electric field maximum is located in the high index material. Additionally, a perturbing beam is mounted on a singly clamped cantilever to tune the cavity. This geometry brings two key benefits relative to previous reports in GaAs systems. First, 1D-PhCCs possess intrinsically low mode volumes, on the order of $\frac{1}{2}{\left(\frac{\lambda }{n}\right)}^{3}$ ,^44^ providing a favorable environment for strong light-matter interactions. Second, the evanescent field of the cavity mode is easily accessible in a side-coupled geometry from both sides of the cavity^45^ allowing for the possibility of simultaneous cavity-waveguide coupling and cavity mode tuning. Such a device could enable waveguide-mediated, cavity–cavity coupling with the integration of single quantum emitters. These systems have recently been shown to have applications as high-efficiency, high-fidelity broadband single-photon switches,^46^ an important component in scaleable photonic circuits.

Our electromechanical approach allows precise, voltage-controllable tuning of the cavity wavelength and the QD emission energy. Here, we present the design and optimization of the cavity and cantilever, with experimental characterization of the cavity tuning behavior. Finally, we demonstrate the capabilities of the device by measuring the cavity mode tuning signal from the device output coupler, highlighting the waveguide coupling capabilities of our device. Additionally, we tune a QD through the cavity mode and measure the resulting Purcell enhancement.

## Results and Discussion

### Device Design

Figure 1 details the design and operational principle of our device. An SEM image of the device is shown in Figure 1a. Each device consists of a singly clamped cantilever terminated by a 1D photonic-crystal (perturbing beam) which, at rest, lies in close proximity to a 1D-PhCC. A bus waveguide is present on the opposite side of the cavity to enable side-coupling to the cavity (see Figure 1b). This waveguide is terminated by a shallow-etched grating output coupler optimized for the QD emission wavelength (900–920 nm). The structures are fabricated in a 170 nm thick GaAs membrane containing self-assembled InAs QDs. The top and bottom layer of the membrane are *p*- and *n*-doped, respectively, enabling the formation of a *p*-*i*-*n* diode across the QD layer and allowing *V*_QD_ to be applied across the QDs. Beneath the membrane, an AlGaAs sacrificial layer is selectively etched after the structures are patterned into the membrane to create free-standing devices. The full wafer structure is shown in Figure 1c. Importantly the substrate, directly under the AlGaAs sacrificial layer, is *n*-doped enabling the creation of a *p*-*i*-*n*-*i*-*n* diode across the whole structure. This allows *V*_EM_ to be applied between the cantilevers and the substrate.

**Figure 1:**
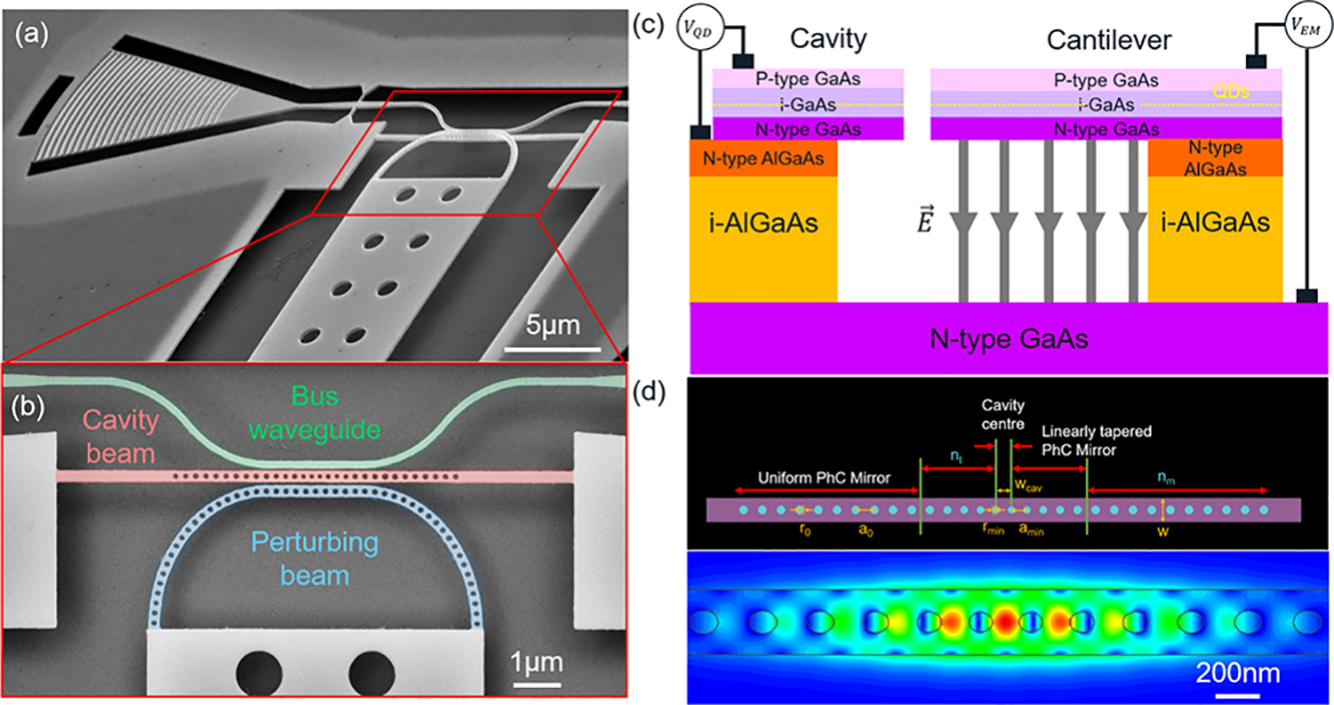
(a) SEM image of an electro-mechanically tunable, waveguide-coupled cavity device. (b) False-color SEM image of the cavity-waveguide interface region. The green, red, and blue sections denote the bus waveguide, cavity beam, and perturbing beam, respectively. (c) Schematic of the wafer and diode structures in the device. Applied voltage *V*_QD_ tunes the QD transition energy and *V*_EM_ moves the cantilever toward the substrate via an electrostatic force. (d) Top: Schematic of the 1D-PhCC design, optimized via FDTD simulations. Bottom: Simulated electric field profile of the cavity’s fundamental resonance.

To isolate the tuning of QDs from the cantilevers, a trench is etched through the *p*-type layer at the base of the cantilever to break the electrical continuity between the cantilevers and the cavities. This design should in principle, allow for the independent tuning of the QD and cavity energies simultaneously. However, due to insufficient isolation between the two diodes, while we demonstrate the tuning of both the QD and cavity energies, we are currently unable to demonstrate this simultaneously (For further details, see text on page S6 and Figure S7 in the Supporting Information). A cantilever size of 35.0 × 7.5 μm is chosen to give the best compromise between a low actuation voltage and resistance to drooping when the sacrificial layer is removed.

A schematic and electric field profile of the 1D-PhCC cavity are shown in Figure 1d. The cavity beam contains three regions: the cavity center with width *c*_w_, a linear taper with minimum pitch *a*_min_, minimum air hole radius *r*_min_ and number of periods *n*_*t*_, and a uniform mirror with pitch *a*, air hole radius *r*_0_ and number of periods *n*_*m*_. The thickness of the nanobeam *t* is fixed by the wafer parameters and the width is chosen to be *w* = 280 nm to support single mode propagation. Each parameter was optimized using FDTD simulations to conduct parameter sweeps for fixed nanobeam dimensions. The optimized cavity design exhibited a Q-factor of *Q* ∼ 5 × 10^6^ without losses and a modal volume of $V_{m}=0.48{\left(\frac{\lambda }{n}\right)}^{3}$ (where *n* = 3.4). The design of the taper region is critical to achieving the optimal Q-factor as it acts to smooth the interface between the cavity and mirror modes which introduces scattering losses.^47,48^ In practice, as we discuss later, the cavity Q-factors are reduced into the range of a few thousand due to the presence of inadvertent losses. In our design, we employ a linear taper where the ratio between the pitch and radius is constant i.e. $\frac{a_{0}}{r_{0}}=\frac{a_{\min}}{r_{\min}}$ . The optimal value obtained from our simulations of *a*_min_ = 0.84*a*_0_ is consistent with previous studies in the literature.^49^

The perturbing beam design was also optimized to minimize scattering losses associated with beam displacement in the evanescent cavity field while achieving a large tuning range. Similarly, the bus waveguide design was optimized to balance between the cavity-waveguide coupling strength, and the cavity Q-factor. This was achieved by carefully adjusting the separation between the cavity and bus waveguide and modifying the waveguide width to optimize the spatial and k-space overlap between the cavity and waveguide modes (see Supporting Information for more detail).

Figure 2 illustrates the operating principle of the device. When *V*_EM_ is applied, an electric field is produced between the cantilever and the substrate. This effectively creates a capacitor with charges accumulating on each of the “plates”. As *V*_EM_ is increased, the electric field strength increases due to the buildup of oppositely signed charges on each plate. At a critical charge accumulation, such that the force of electrostatic attraction is greater than the restoring force produced by the elastic strain of the cantilever, the suspended structure will begin to deflect toward the substrate. As the cantilever actuates, the perturbing beam gradually shifts out of the evanescent cavity mode. With increasing deflection, the influence of the perturbing beam on the cavity mode index diminishes, thus, reducing the effective mode index resulting in a blue-shift of the cavity mode.

**Figure 2:**
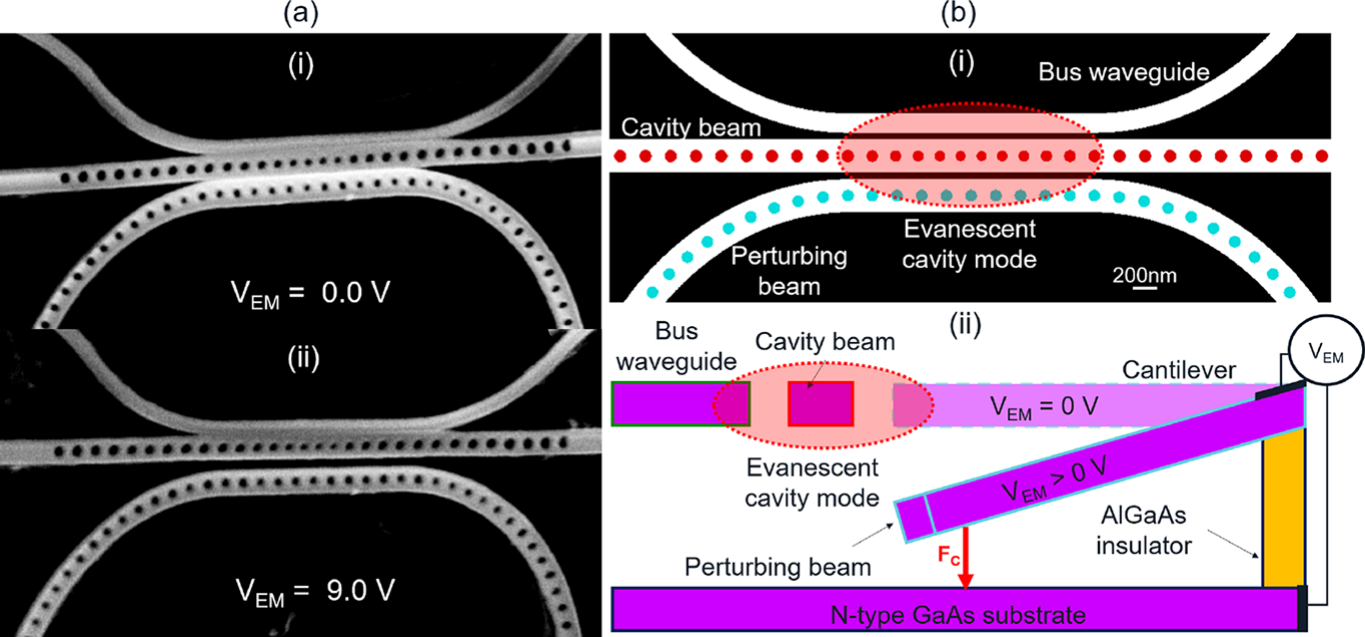
(a) Angled SEM images of cavity/bus waveguide region at (i) *V*_EM_ = 0.0 V and (ii) 9.0 V showing the downward displacement of the perturbing beam at 9.0 V due to the electrostatic actuation of the cantilever. (b) (i) Illustration of the spatial overlap between the evanescent cavity mode and the perturbing beam, which is actuated to tune the cavity wavelength. (ii) Schematic of device operation depicting the application of bias between the cantilever and the substrate creating an electrostatic force which deflects the cantilever toward the substrate.

The displacement of the cantilever is governed by the following relationship:^50^

$$\left(d_{0}-d\right)=\frac{2\epsilon L^{4}}{Ed^{2}t^{3}}V^{2}$$ 1

Where *V* is the applied bias, *d*_0_ is the distance between the cantilever and the substrate at equilibrium (*V* = 0), *d* is the displacement, ϵ is the permittivity of the material between the substrate and the membrane, *L* is the length of the cantilever and *E* is the Young’s modulus of the cantilever material. The maximum displacement of the perturbing beam from the equilibrium point is therefore reached at the pull-in voltage *V*_pi_, the point where the electrostatic and restoring forces are balanced. This corresponds to a displacement of $d_{\max}=\frac{1}{3}T_{\mathrm{SL}}$ where *T*_SL_ is the thickness of the sacrificial layer. Above *V*_pi_, the electrostatic force overcomes the restoring force, causing the cantilever to collapse and adhere to the substrate. If *V*_EM_ is reduced to zero, the cantilever can recover to its initial state if the restoring force is greater than the van der Waals force between the substrate and the cantilever. Using eq 1, we calculate the theoretical pull-in voltage of our devices to be *V*_pi_ = 2.50 V. Figure 2ai,ii, shows angled SEM images taken at room temperature of a device at *V*_EM_ = 0.0 and 9.0 V respectively, where the displacement of the perturbing beam relative to the cavity beam is evident. Notably, the beam does not collapse at 9.0 V despite this voltage exceeding the theoretical pull-in voltage, which we attribute to resistance in the circuit used to bias the cantilever. The actuation voltage is reduced when operating at cryogenic temperatures due to the reduced resistance. At cryogenic temperatures, the pull-in voltage becomes *V*_pi_ ∼ 6.5 V.

We present the results from three tunable cavity devices (labeled device 1,2 and 3, respectively) to best show the capabilities of our design. Devices 1 and 3 share design parameters whereas device 2 has slightly adjusted parameters to improve the cavity mode uniformity and cavity-waveguide coupling efficiency. Namely, *a*_min_ = 184 nm, *w*_bus_ = 180 nm for devices 1 and 3 and *a*_min_ = 188 nm, *w*_bus_ = 190 nm for device 2. Device 2 also uses a deep-etched grating coupler design as opposed to the shallow-etched design used in devices 1 and 3 to simplify the fabrication process by reducing the required number of etch steps. The main factor for the difference in the performance of the three devices is fabrication imperfections causing unintended fluctuations of the device parameters. Mitigating the effect of fabrication imperfections remains a significant challenge in the development of scaleable photonic circuits incorporating QDs. Recent improvements in fabrication procedures such as surface passivation^51^ wet etching^52^ and resist reflow^53^ techniques show the potential to improve the performance and uniformity of nanostructures by reducing absorption and scattering from the surfaces of the devices.

### Cavity Tuning Characteristics

To study the tuning behavior of the structures, the wavelength and Q-factor of the cavity mode were measured as a function of *V*_EM_ using a micro-photoluminescence (μ-PL) setup. An above-band laser (808 nm) was used to excite the broadband QD ensemble in the cavity region, thereby illuminating the cavity mode. The displacement of the perturbing beam was estimated using eq 1, with a small empirical offset in voltage of *V*_off_ = 3.8 V to account for the circuit resistance. Figure 3 shows a comparison between the experimentally measured and simulated tuning behavior of a typical cavity (device 1). Overall, a good agreement between the simulated and experimentally measured structures can be seen for both the Q-factor (Figure 3a,c) and wavelength dependence (Figure 3b,d) as a function of displacement. To reproduce the lower Q-factors seen experimentally compared to the optimized simulated cavities - which exhibit Q-factors in excess of 1 × 10^6^ - we introduce large scattering losses from the cavity and perturbing beams by modulating the size and positions of the air holes within the beams. This reduces the Q-factor of the simulated structures to the same order of magnitude as the measured devices. The Q-factor increases slowly at displacements between 0 and 50 nm, before increasing more rapidly at larger displacements above 50 nm. The relationship between the Q-factor and the displacement becomes roughly linear in the simulation at displacements larger than 150 nm. The overall increase in the Q-factor is smaller in the experimental data compared to the simulated device. This may be due to the lower starting Q-factor in the experimental device. As the Q-factor continues to increase right up to the end of the range, this implies that scattering losses from the perturbing beam are the dominant loss mechanism (see Supporting Information for more detail). These are likely due to suboptimal air hole radii in the perturbing beam and sidewall roughness in the fabricated device. In Figure 3b a small red-shift in the cavity wavelength is observed at small perturbing beam displacements. This behavior is also seen in the simulations, although the magnitude of the red-shift is larger in the simulated device. This shift may be due to the initial displacement shifting the perturbing beam through an antinode of the cavity mode field, thus increasing the effective index of the mode and producing a red-shift in the cavity mode wavelength. The maximum observed tuning range of this device was Δλ = 0.55 nm. This range corresponds to an in-plane separation of approximately 85 nm between the cavity and perturbing beams in simulation. This agrees with the typical in-plane separation extracted from SEM images of the fabricated devices (see Supporting Information for further details).

**Figure 3:**
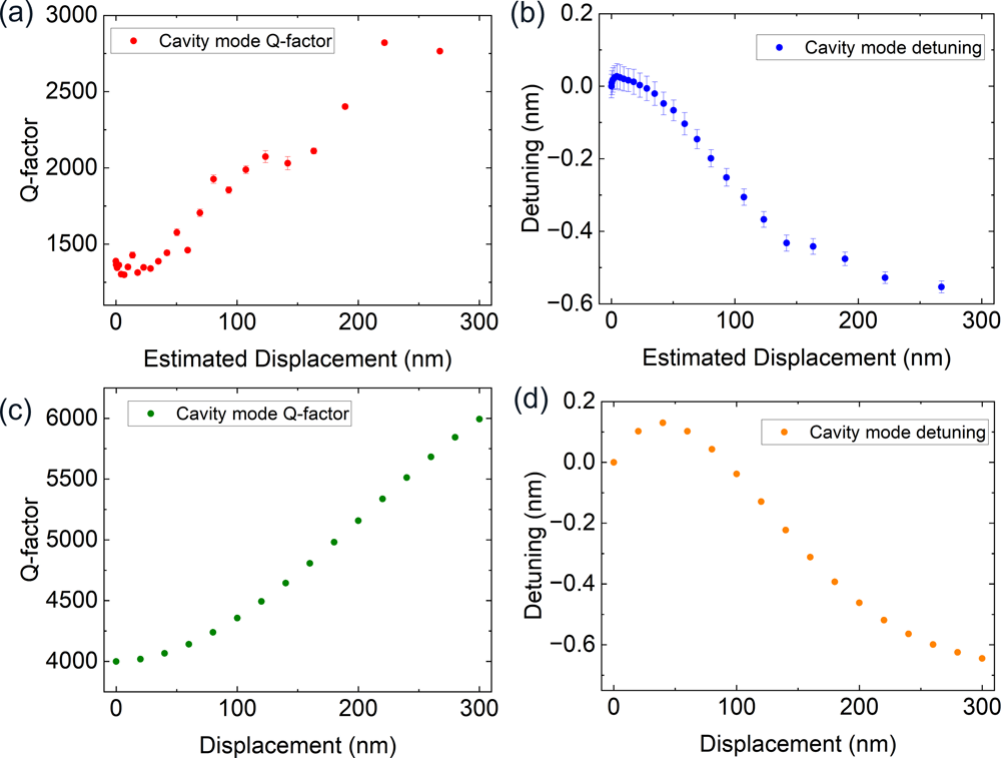
Device 1. (a,c) Experimental and simulated relationship between Q-factor and perturbing beam displacement, respectively (b,d) Experimental and simulated relationship between cavity mode detuning relative to initial cavity wavelength at *V* = 0 and perturbing beam displacement, respectively.

To demonstrate bus waveguide coupling, the cavity detuning characteristics of a second cavity (device 2) were measured by exciting the cavity mode with an above-band laser and collecting the light scattered out of the structure by the output coupler which terminates the bus waveguide as shown in Figure 4a. Figure 4b shows the PL spectra of the cavity mode with the cantilever displaced by 0 nm (black) and 300 nm (red), using the excitation and collection scheme depicted in Figure 4a. Device 2 exhibits the same blue-shifting behavior as observed for device 1. However, the maximum detuning observed from this device was larger at Δλ = 1.8 nm with the detuning vs displacement dependence being shown in Figure 4c. This tuning range corresponds to a separation of approximately 57.5 nm between the perturbing and cavity beams, which is estimated from the simulated relationship between detuning range and in-plane separation between the cavity and perturbing beams (see Supporting Information for further details). The difference in separation between device 1 and device 2 is a result of slight variations in the design parameters. This large difference in tuning range (0.55 nm vs 1.8 nm) demonstrates the sensitivity of the device to the in-plane separation between the beams (85 nm vs 57.5 nm). We also observe the expected increase in the Q-factor of the cavity mode as the cantilever is deflected as seen in the first device, increasing from *Q*_0_ = 640 at a displacement of 0 nm, to *Q*_300_ = 1530 at a displacement of 300 nm, similar to the behavior observed in Figure 3a,c. The results from Figures 3 and 4 clearly demonstrate the robust, voltage-controllable cavity mode wavelength tuning capabilities of our device and how the light can be extracted off-chip via a side-coupled bus waveguide terminating in a grating output coupler. Furthermore, the indirect cavity tuning does not significantly degrade the Q-factor of the cavity. Our devices therefore exhibit key attributes necessary for the effective scale-up of QD-cavity based systems.

**Figure 4:**
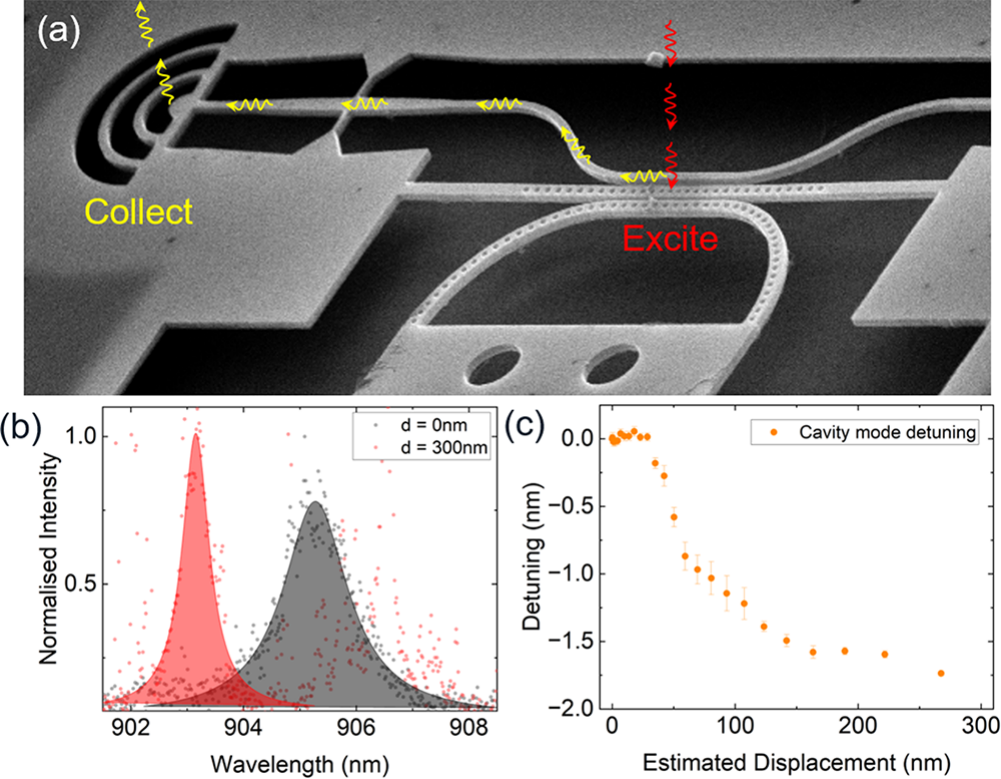
Device 2. (a) Annotated SEM image detailing the excitation and collection scheme of the measurements in (b) and (c). (b) Cavity mode PL spectra of device 2 with perturbing beam displacements of 0 nm (gray) and 300 nm (red). The additional sharp lines arise due to emission from QDs in the outcoupler which are excited by scattered laser light. (c) Experimentally measured relationship between the cavity mode detuning and the perturbing beam displacement for device 2 demonstrating a maximum tuning range of 1.8 nm.

### Quantum Dot Tuning into Resonance with Cavity

To demonstrate further the potential of our device for quantum technologies applications, we explored the behavior of a single QD located in a third cavity (device 3) from the same wafer. Figure 5a shows the PL spectrum of a QD state tuning as a function of the applied bias when exciting and collecting directly above the cavity. Here, a bias is applied between the top of the device membrane and the top of the device substrate, forming a *p*-*i*-*n*-*i*-*n* diode. As the current flow through the *p*-*i*-*n*-*i*-*n* is lower than in a *p*-*i*-*n* for a given voltage, a larger voltage can be applied across the QDs than in a *p*-*i*-*n* structure. This enables us to observe quantum-confined Stark-tuning of the QDs in the voltage range 2–5 V as shown in Figure 5a. A clear enhancement in emission intensity is observed when the QD becomes degenerate with a weakly excited cavity mode at ∼902.5 nm, a signature of Purcell enhancement. Furthermore, a single PL spectrum corresponding to the orange shaded region in Figure 5a is plotted in Figure 5b. This shows the QD PL signal (red curve) with the cavity mode (green curve), as distinct spectral features. The apparent splitting of the cavity mode arises due to interference fringes in our experimental setup originating from a Fabry–Pérot cavity formed between the membrane and the substrate in the wafer. By filtering for the QD emission only and removing the influence of the near-resonant cavity mode, we investigated the photon statistics of the QD, represented by *g* ^(2)^(τ). As shown in Figure 5c, a clear antibunching dip at zero delay time (τ = 0) is observed, with *g* ^(2)^(0) = 0.176 ± 0.142, well below the threshold of 50% for single-photon emission. Figure 5d further explores the interaction between the QD and the cavity by plotting the nonresonant PL lifetime of the QD emission as a function of detuning from the cavity mode. A substantial reduction in radiative lifetime is observed when the QD is in resonance with the cavity, reaching a minimum of approximately 350 ps at zero detuning (limited by the APD response time). This imposes a lower bound on the Purcell factor of the QD of *F*_P_ = 3.5.

**Figure 5:**
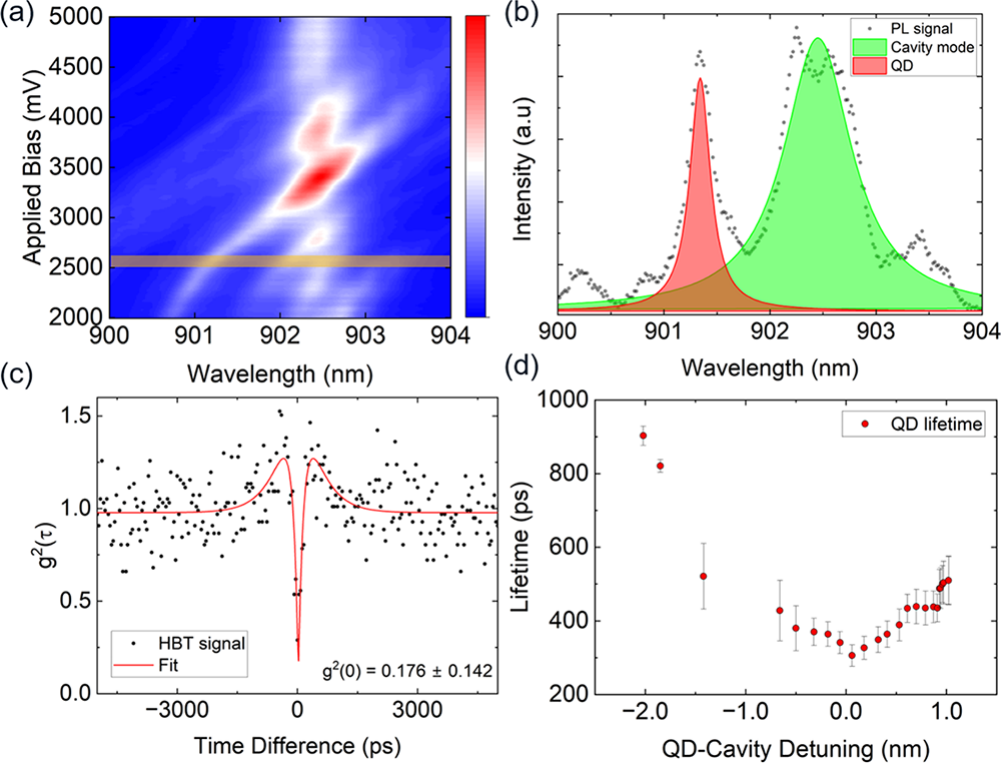
Device 3. (a) QD emission line electrically tuned via Stark tuning through a cavity mode ∼902.5 nm. (b) Single QD PL-spectrum from (a) (shaded orange region). (c) Second-order correlation measurement of the emission from the QD in (b). (d) Nonresonant PL lifetime of the QD as a function of cavity detuning.

## Conclusions

This work demonstrates the successful integration of quantum dots (QDs) with tunable photonic-crystal cavities on a GaAs platform, enabling precise control over QD-cavity coupling. By combining voltage-controlled QD tuning with cavity mode adjustment via electro-mechanically actuated cantilevers, we achieve robust and repeatable resonance alignment between QD emission and single cavity modes. This tuning mitigates the challenges posed by the stochastic nature of QD positioning and fabrication-induced variations in cavity dimensions—a key barrier to scalable QD-cavity coupled systems.

The good agreement between simulated and experimentally measured tunable cavity Q-factors and wavelengths provides a reliable framework for realizing this design as a scalable platform for multiple cavity-coupled systems. Furthermore, the device design, employing a 1D-PhCC with a low mode volume, ensures efficient coupling between QDs and cavity modes while minimizing losses. The implementation of cantilever-based tuning offers a localized method for cavity wavelength adjustment, preserving the intrinsic optical properties of the QDs — a challenge in many other systems.

The experimental results highlight several key achievements. First, a large, voltage controllable cavity mode tuning range is observed in a side-coupled geometry. This paves the way for scale-up to multiple waveguide-mediated coupled-cavity devices, with efficient on and off-chip coupling of photons. Second, Purcell enhancement is observed through increased PL intensity and reduced PL lifetime when QDs are resonant with cavity modes. Additionally, single-photon emission with marked antibunching characteristics is demonstrated, with photon purity limited only by material losses and nonresonant excitation conditions. Finally, we achieve such strong single-photon behavior within a tunable cavity system, enabling more intricate studies of QD-cavity interactions in future work.

Overall, this work establishes a robust framework for achieving precise QD-cavity coupling in semiconductor systems, paving the way for advancements in quantum light sources and cavity-enhanced photon emission. These results lay the foundation for future exploration of complex quantum systems, including multi-QD cavity-coupled systems for the realization of cavity-based optical switches in photonic circuit designs and the investigation of QD–QD interactions within coupled-cavity environments.

## Methods

The wafer was grown on a (100) GaAs substrate via molecular beam epitaxy. The top 300 nm of the substrate is *n*-doped with silicon atoms at a density of 2 × 10^18^ cm^–2^. A 1.15 μm layer of Al_0.6_Ga_0.4_As is grown on top of the substrate, the top 200 nm of which is *n*-doped with silicon atoms at a density of 2 × 10^18^ cm^–2^. This sacrificial layer is selectively etched away in the device fabrication process. Next, the device membrane containing embedded InAs QDs is grown. The primary material of the 170 nm thick layer is GaAs, in addition, two Al_0.3_Ga_0.7_As quantum well barrier layers are included either side of the QD layer to increase the electrical tuning range of the QD emission energies. The barrier above/below the QD layer is 30/50 nm thick, respectively. The QDs are grown by the Stranski–Krastanov method and are located at the center of the membrane. The top/bottom 30 nm of the membrane are *p-*/*n*-doped with carbon/silicon atoms at a density of 2 × 10^19^ cm^–2^ and 2 × 10^18^ cm^–2^, respectively.

The nanostructures were patterned into a SiO_2_ resist via electron beam lithography and were subsequently transferred into the membrane through an inductively coupled plasma etch. The wafer was then exposed to HF acid to selectively etch away the sacrificial layer and produce free-standing structures. Finally, the wafer underwent a critical-point dry process to preserve the structures during the removal of the residual acid. To create the diode structures as depicted in Figure 1c mesas were etched into the wafer at a depth of 150 nm for the QD tuning and 1300 nm for the cantilever actuation. The *p-* and *n*-type layers were then patterned with Ti/Au contacts which were connected to individual pins on the chip carrier to enable separate bias’ to be applied to the QD and cantilever layers.

The sample was placed in a low pressure helium exchange-gas bath cryostat on 3-axis piezoelectric stages. A pair of achromatic doublets and an aspheric lens are used to efficiently focus light onto and collect emission from the sample.

The optical measurements were conducted using a standard confocal micro-photoluminescence setup where the QDs and cavity modes were excited nonresonantly with an above band (808 nm) continuous-wave laser. The spectra were recorded using a silicon charge-coupled detector positioned after a grating spectrometer. The time-resolved data shown in Figure 5d was obtained via excitation from a pulsed Ti:S laser operating above band (∼810 nm) at ∼80 MHz repetition rate. The signal was detected using an APD with a timing resolution of 350 ps and dead time of 22 ns connected to a time tagger with a bin width of 50 ps.

## Supplementary Material



## Notes

Details of optimization of cavity design, tolerance of cavity design to fabrication disorder, details of electrical isolation, PL map of QD and cavity tuning in a single device, simulated tuning range of device, effect of material loss on cavity performance, evolution of cavity mode in Figure b, side-coupling efficiency, schematic of the setup and details of the cross-correlation measurement, and details of time-resolved measurements (PDF)
