# Supplementary material for: Electro-Mechanically Tunable, Waveguide-Coupled Photonic-Crystal Cavities with Embedded Quantum Dots
Source: ACS Photonics. 2025 Jul 17;12(8):4315–22. doi: 10.1021/acsphotonics.5c00606 (PMC12372170; doi:10.1021/acsphotonics.5c00606)
Supplement: Supplementary file 1 [file ph5c00606_si_001.pdf]

Supporting Information - Electro-Mechanically Tunable, Waveguide-Coupled Photonic-Crystal Cavities with Embedded Quantum Dots

L. A. F. Brunswick,*,† L. Hallacy,† R. Dost,† E. Clarke,‡ M. S. Skolnick,† and L. R. Wilson†

†*School of Mathematical and Physical Sciences, University of Sheffield, Sheffield, S3 7RH, UK*

‡*School of Electrical and Electronic Engineering, University of Sheffield, Sheffield, S3 7HQ UK*

E-mail: l.brunswick@sheffield.ac.uk

Number of figures: 15

Number of tables: 2

Number of pages: 16

Nanobeam Cavity Optimisation

This section will contain details of the simulation study performed to optimise the design of the 1D photonic-crystal cavity (PhCC) used in the main text. All data is reproduced from.[1]

Uncoupled, Lossless Cavity Optimisation

The design of the 1D-PhCC was optimised through a set of parameter sweeps conducted in Lumerical 3D-FDTD simulations. Each of the parameters from Figure 1 (d) in the main text, were swept over a large range while the other parameters were held constant.

The two most sensitive parameters in the design were the minimum period of the photonic-crystal (a_{min}) and the cavity width (c_w). The results from the parameter sweeps of a_{min} and c_w are shown in Figure S1 and Figure S2, respectively. Both parameters exhibit a strong resonance-like behaviour about the optimal value in their Q-factor dependence, demonstrating the sensitivity of these parameters. This is especially true of a_{min}, as fabrication imperfections largely manifest in air hole radii fluctuations, rather than positional errors.

Further to this, the parameters of the photonic-crystal in the perturbing beam were optimised to reduce losses associated with the presence of the perturbing beam in the evanescent cavity field. From Figure S3, we can see that the reduction in Q-factor caused by the actuation of the perturbing beam can be eliminated by using the optimal photonic-crystal period. A value of $a_p = 210$ nm was found to be optimal in our simulations. This is slightly smaller than the value of $a = 220$ nm used for the cavity beams. This reduction in the period does not significantly alter the achievable tuning range of the device as shown by Figure S3 (b).

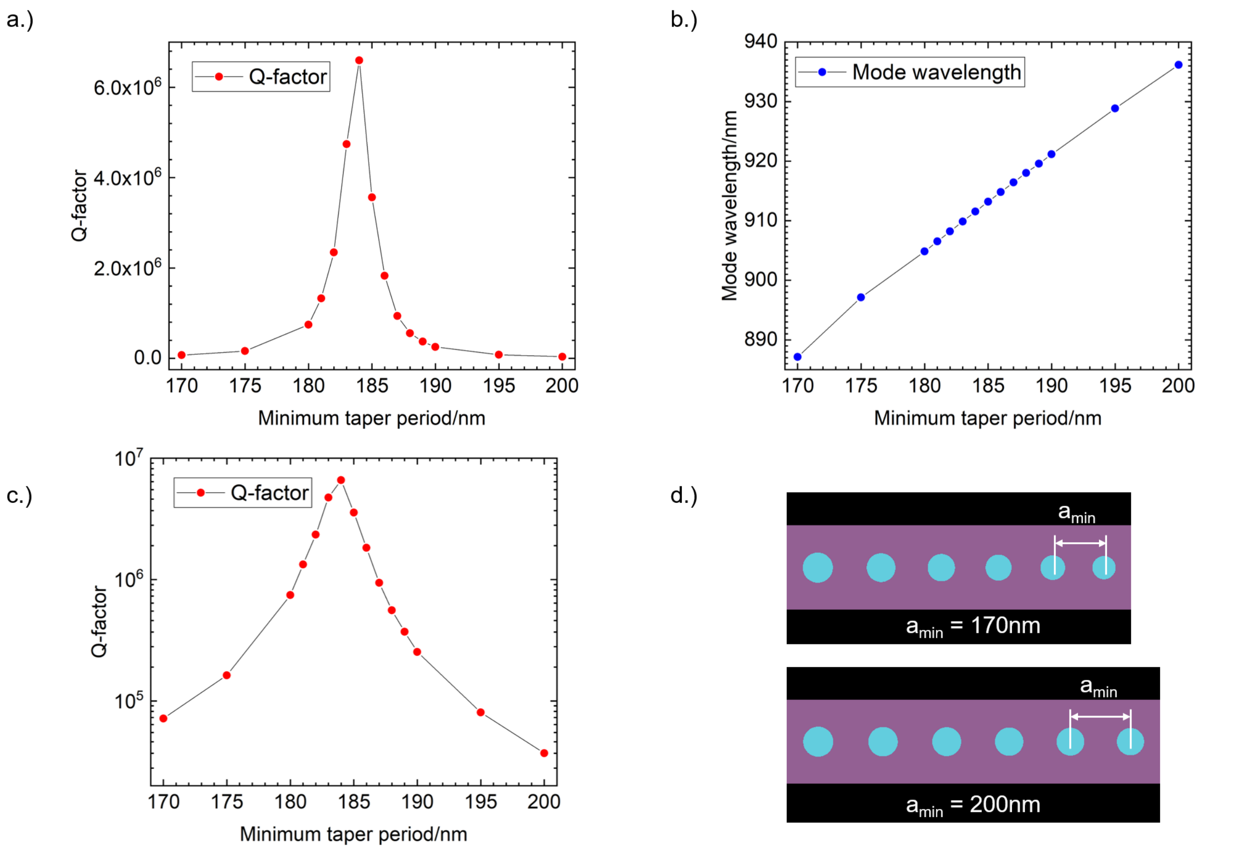

Figure S1: Results from the parameter sweep simulations of a_{min} for (a) Q-factor and (b) cavity mode wavelength. (c) Q-factor result presented on a logarithmic scale. (d) Visualisation of the effect on the structure of changing a_{min}.

Nanobeam Cavity Fabrication Tolerance

To assess how robust to fabrication imperfections the optimised cavity design was, a Monte Carlo simulation study was conducted for three different air hole radii errors: 2%, 5% and 10%. In each case, 50 different devices were simulated where the radius of each air hole was modified randomly within a uniform distribution. Figure S4 (a) and (b) show the effect of the three different levels of fabrication disorder on the Q-factor and cavity mode wavelength, respectively. Table 1 shows the numerical results from the Monte Carlo simulations. As the level of error increases, the minimum and median Q-factor values reduce, while the standard deviation in the cavity mode wavelength distribution increases.

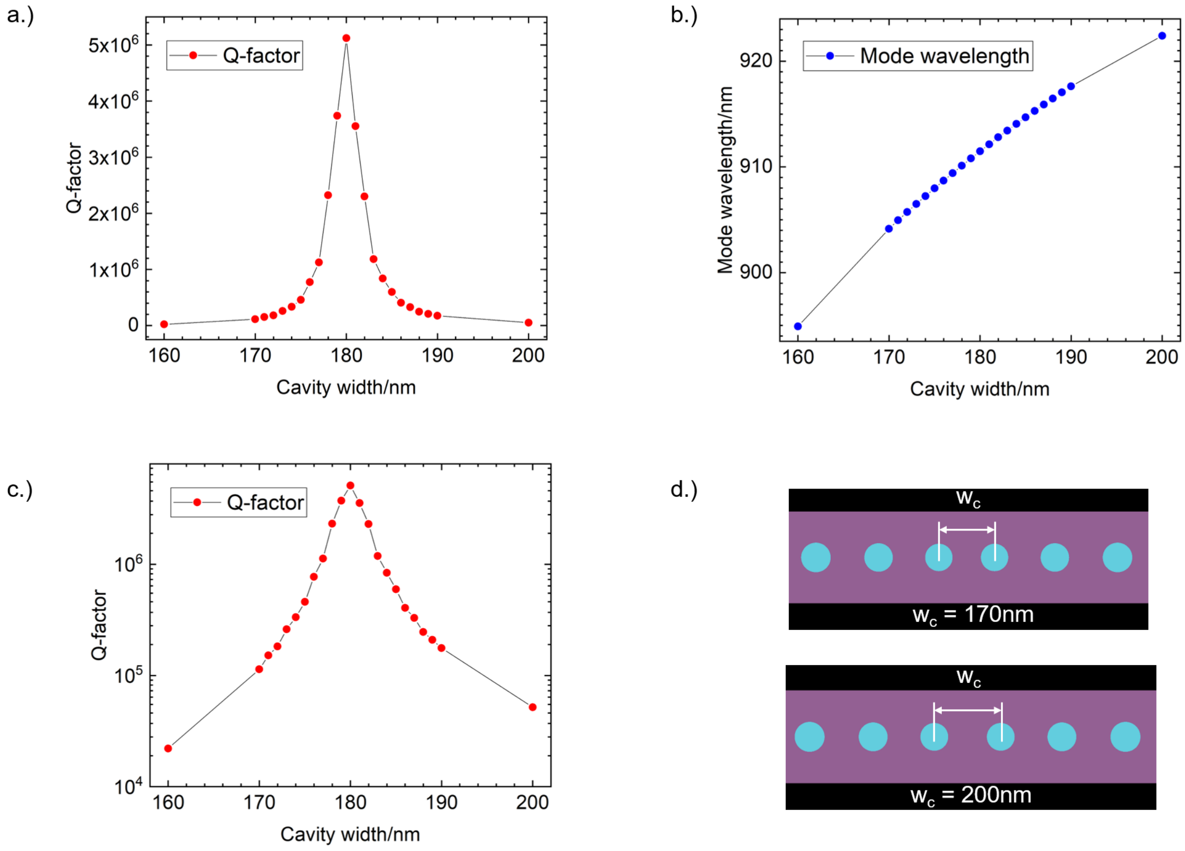

Figure S2: Results from the parameter sweep simulations of c_w for (a) Q-factor and (b) cavity mode wavelength. (c) Q-factor result presented on a logarithmic scale. (d) Visualisation of the effect on the structure of changing c_w.

Table 1: Qualitative results from Figure S4

**Error**	Q_{Min}	Q_{Med}	σ_λ
2%	1.26×10^6	2.20×10^6	0.29 nm
5%	2.69×10^5	5.56×10^5	0.74 nm
10%	7.47×10^4	1.82×10^5	1.99 nm

Prior to fabricating a sample combining the cavity, perturbing beam and waveguide elements into a single device, a sample containing just cavity devices was made. A range of different cavity parameter sets were present on the sample to discern which parameter set gave the best cavity performance in experiment. Figure S5 shows the distribution of cavity mode wavelengths from the parameter set which exhibited the lowest standard

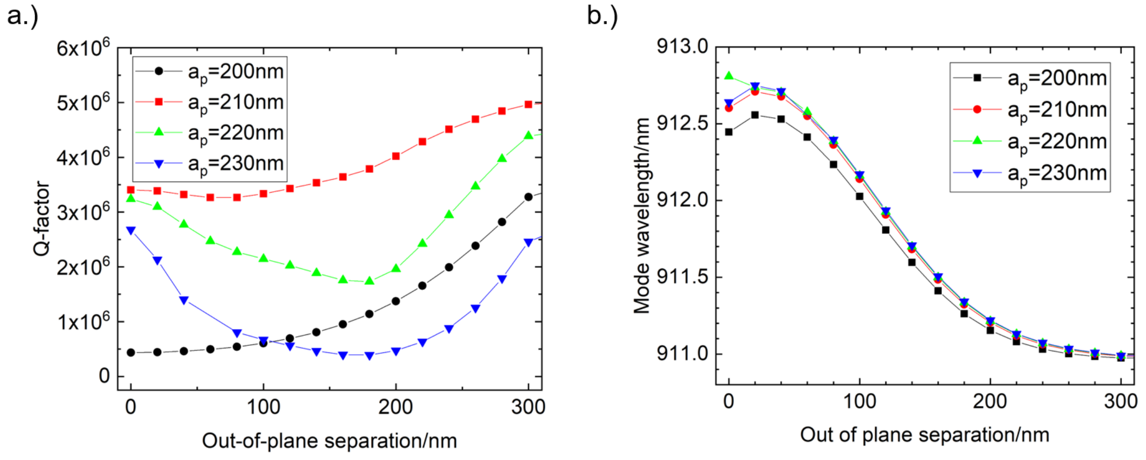

Figure S3: Results from the parameter sweep simulations of a_p for (a) Q-factor and (b) cavity mode wavelength dependence on out-of-plane separation between the cavity and perturbing beam.

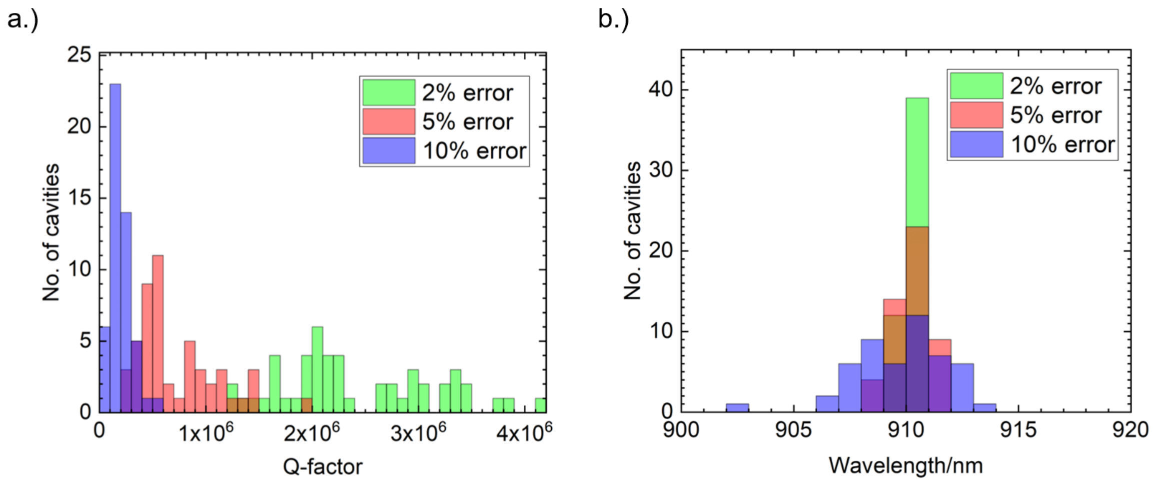

Figure S4: Results from the Monte Carlo simulations for 2% (green), 5% (red) and 10% (blue) error on the air hole radii.

deviation of $\sigma_\lambda = 0.6$ nm, equating to an error of between 2-5% on the air hole radius.

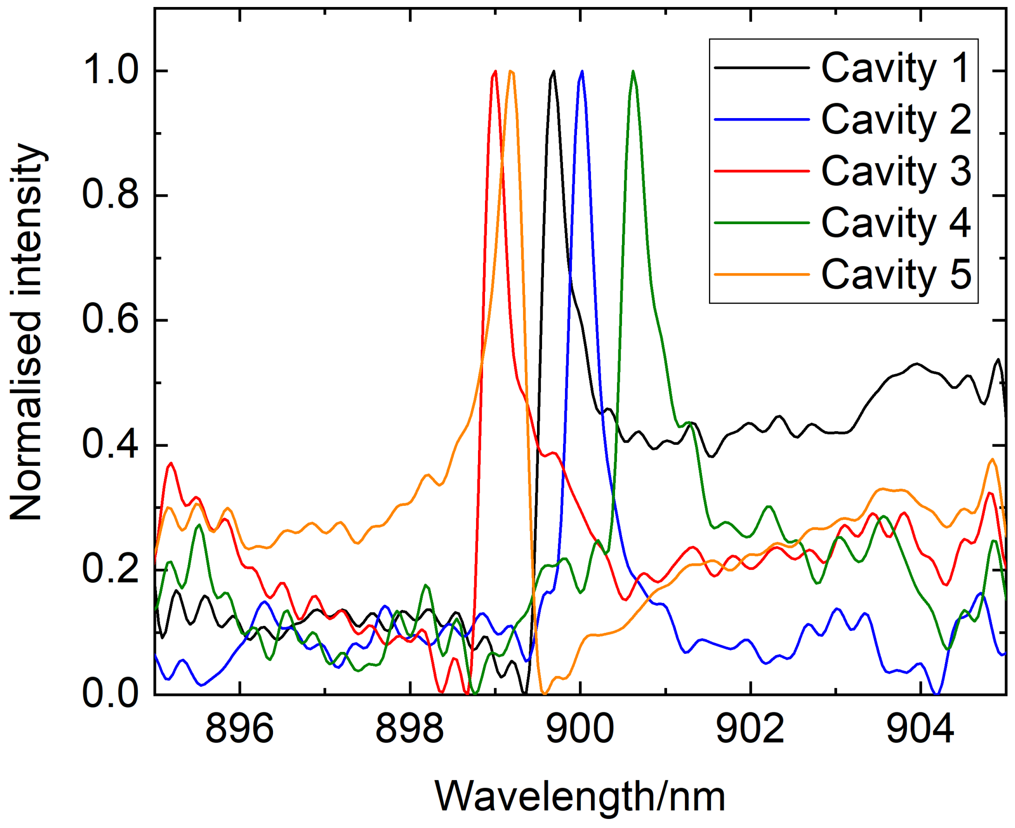

Figure S5: Overlapped white light reflection spectra from the set of like cavities with the lowest standard deviation in cavity mode wavelength distribution.

1D-PhCC Cavity Tuning

Electrical Isolation

Unfortunately, due to electrical cross-talk between the diodes, we were unable to tune both the cavity and QD emission simultaneously in a controllable manner. We attribute this cross-talk to the insufficient depth of the etched isolation trenches in the device membrane. The cross-talk could be eliminated by increasing the depth of the isolation trenches to fully etch through the membrane, rather than just the *p*-type layer. Figure S6 demonstrates that the cross talk between the diodes can be mitigated by grounding the Stark-tuning diode, allowing the cavity energy to be tuned independently of the QD energy.

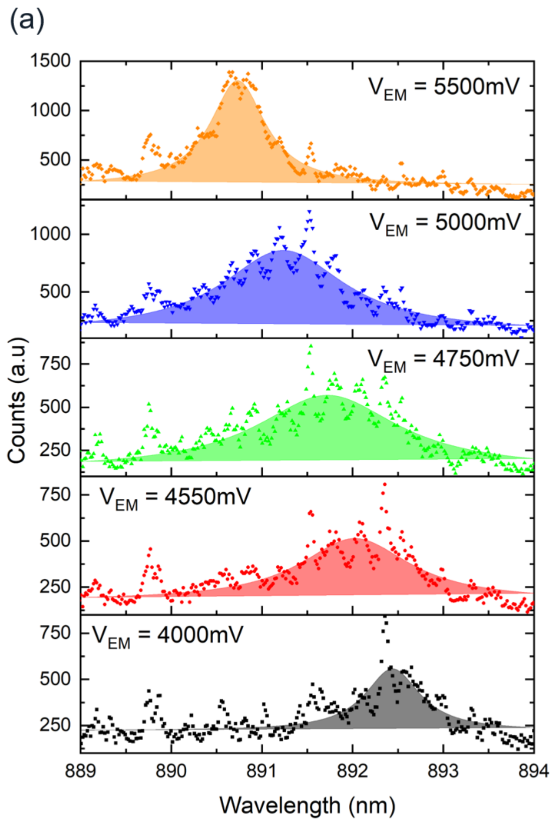

Figure S6: (a) Spectra of a cavity mode showing the tuning due to an applied bias V_{EM} from a starting wavelength $\lambda_{cav} = 892.54$ nm to $\lambda_{cav} = 890.80$ nm. (b) the same cavity mode tuning with QD lines visible at longer wavelengths. The QD lines do not tune significantly over the range of the cavity mode tuning.

QD and Cavity Tuning in a Single Device

While the independent tuning of the QD and cavity emission is not yet observed in our devices, it is possible to observe the tuning of both energies within a single device. Figure S7 shows a PL map of a device where the tuning of both the QD and cavity emission can be seen when applying a bias using V_{EM}.

Tuning Range

The maximum achievable tuning range of our device is strongly dependent on the separation between the cavity and perturbing beams. To quantify this, a simulation study was

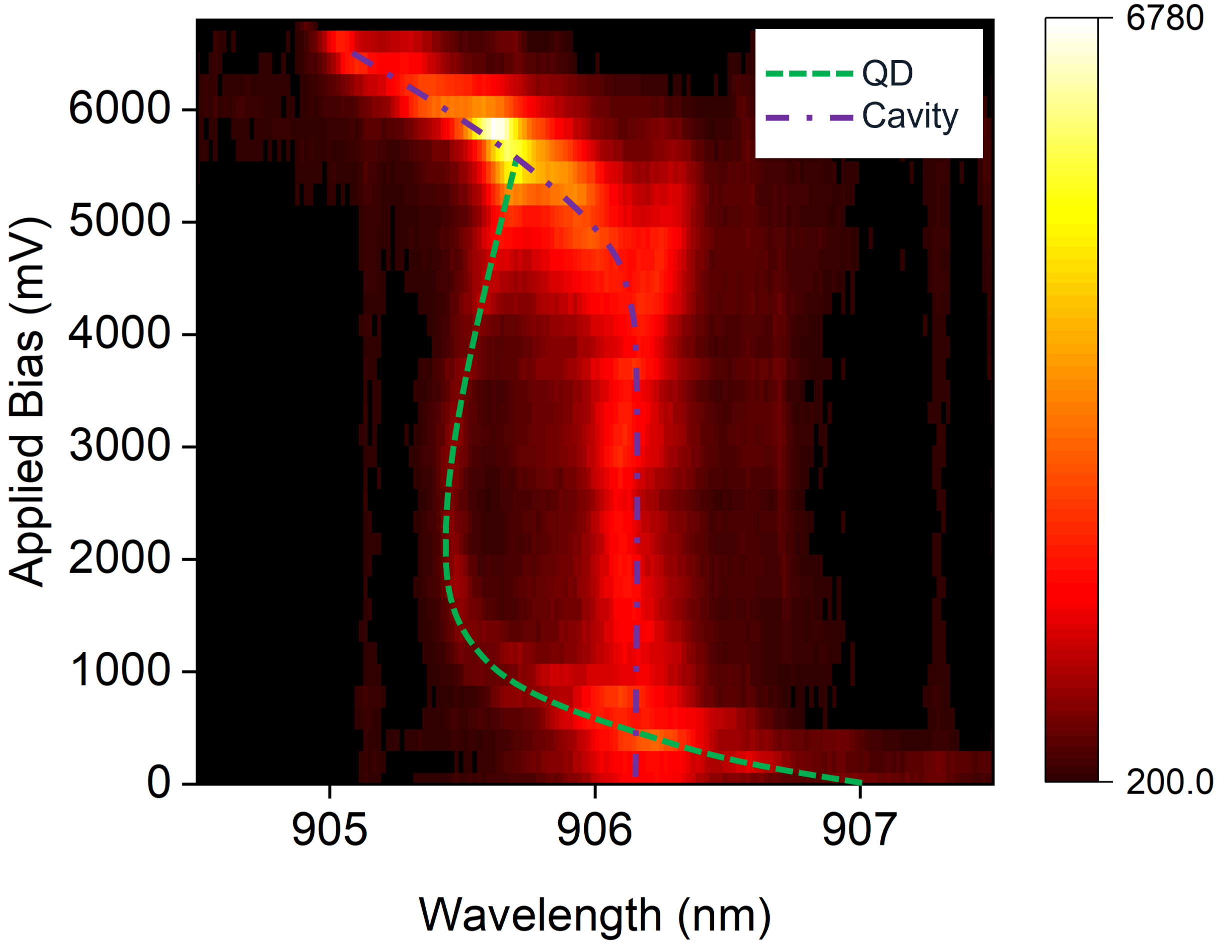

Figure S7: Color-scale PL map showing the tuning of both the QD (green dash line) and cavity (purple dash-dot line) energies in a single device. The cross talk between the QD and cantilever contacts in this device allows both the QD and cavity to be tuned using a single applied bias (V_{EM}). The green and purple lines are added to assist in ease of identification of the QD and cavity lines as the bias is varied.

conducted, the results of which are presented in Figure S8. The maximum tuning range of the device falls off exponentially as the separation between the cavity and perturbing beam increases up until a separation of $\sim$ 130 nm. After this, the cavity mode wavelength no longer blue-shifts as the cantilever is deflected and instead, red-shifts a small amount. The Q-factor dip decreases in a roughly exponential manner as the separation increases. Figure S9 shows an SEM image of a typical structure from the measured sample. The measured separation between the cavity and perturbing beams is $\sim$ 85 nm, which corresponds to a simulated tuning range of 0.55 nm. This is consistent with the results in Figure 4.

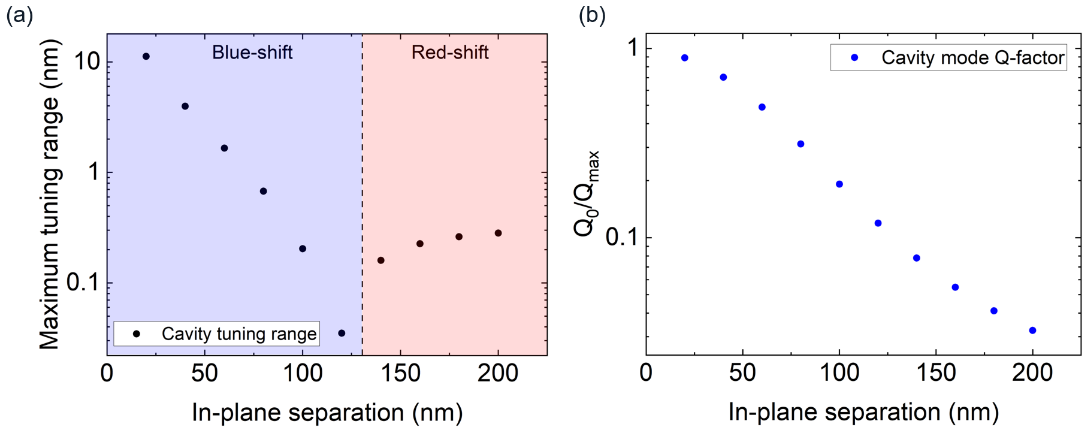

Figure S8: (a) Simulated relationship between the separation between the cavity and perturbing beam and the maximum tuning range of the cavity mode wavelength. (b) Simulated relationship between the Q-factor dip and the separation between the cavity and perturbing beam

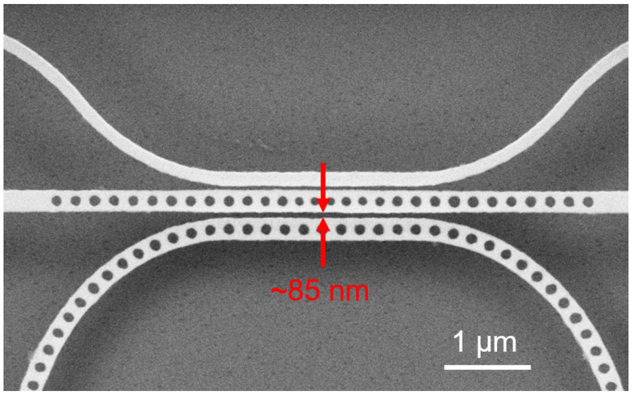

Figure S9: SEM image showing a typical cavity/waveguide/perturbing beam interface with the separation between the perturbing and cavity beam measured to be $\sim$ 85nm.

Effect of Material Loss on Q-factor

To study the effect of material absorption loss on the Q-factor of the cavity, the simulations used to produce Figure 3 (c) & (d) in the main text were repeated with the imaginary refractive index of the material set to $n_i = 1 \times 10^{-4}$ and $n_i = 5 \times 10^{-4}$. Figure S10 (a) and (b) show the relationship between the perturbing beam displacement and the relative and absolute cavity Q-factor, respectively. As the value of n_i increases, both the starting Q-factor (Q_0) and the relative increase in Q-factor decrease. The Q-factor trend also changes, starting to plateau more noticeably at higher values of n_i. We can infer from this, that the

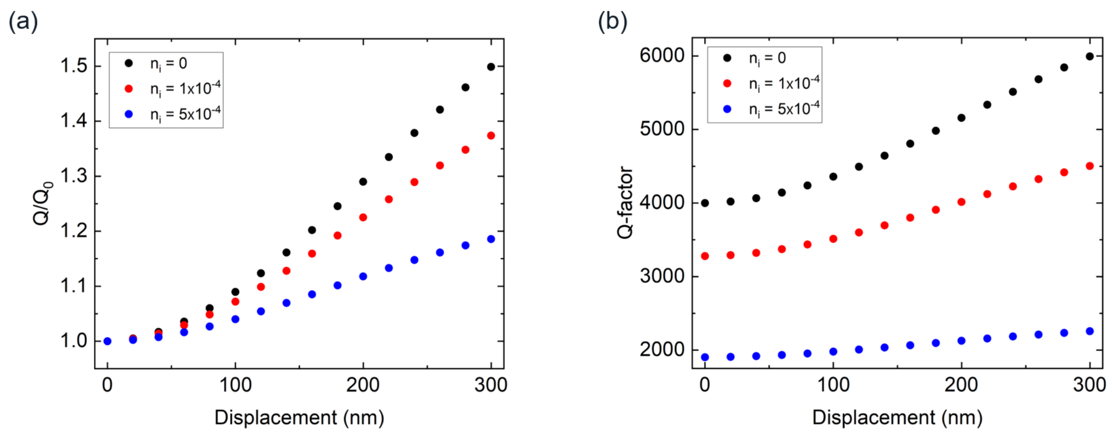

Figure S10: Relationship between the perturbing beam displacement and the (a) relative, (b) absolute, cavity Q-factor for an imaginary refractive index of $n_i = 0$ (black circles), $n_i = 1 \times 10^{-4}$ (red circles) and $n_i = 5 \times 10^{-4}$ (blue circles).

main source of loss in our fabricated device is scattering loss due to the presence of the perturbing beam in the evanescent cavity field.

Figure 4 (b) Tuning Maps

Figure S11 shows the evolution of the cavity mode from device 2 shown in Figure 4. Here we present spectra measured from above the cavity to eliminate the background from QD emission in the outcoupler. The slight shift in the starting wavelength of the cavity mode between the two measurements is due to the different laser power and alignment used to obtain both measurements. The feature of interest is visible when measuring in both geometries confirming that it originates from the cavity region of the device. We see a clear evolution of the cavity mode wavelength as the cantilever is displaced. We also observe the characteristic reduction in Q-factor at intermediate values of displacement.

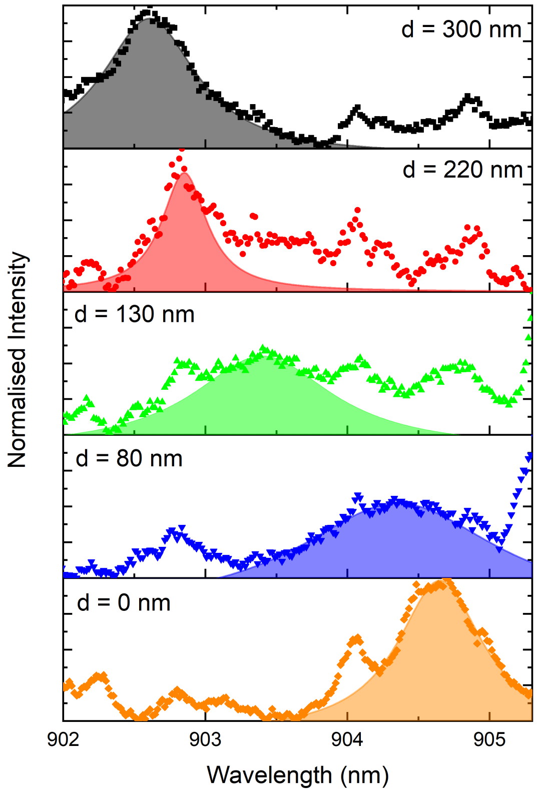

Figure S11: Evolution of the cavity mode spectrum for device 2 shown in Figure 4 with increasing displacement of the cantilever. The spectra presented here are recorded from above the cavity.

Bus Waveguide Coupling

The devices simulated in this section were based on an optimised, lossless cavity with a starting $Q \sim 5 \times 10^6$. Figure S12 (a) and (b) show the relationship between the width of the bus waveguide and the transmission through the device and cavity mode Q-factor for a fixed coupling gap, respectively. The magnitude of the transmission dip increases dramatically in between widths of 180 and 190 nm. This is due to the increased k-space overlap of the cavity and waveguide modes increasing the coupling efficiency between the two modes. This increase in coupling efficiency is also reflected in the reduction of the cavity Q-factor by 3 orders of magnitude over the same range.

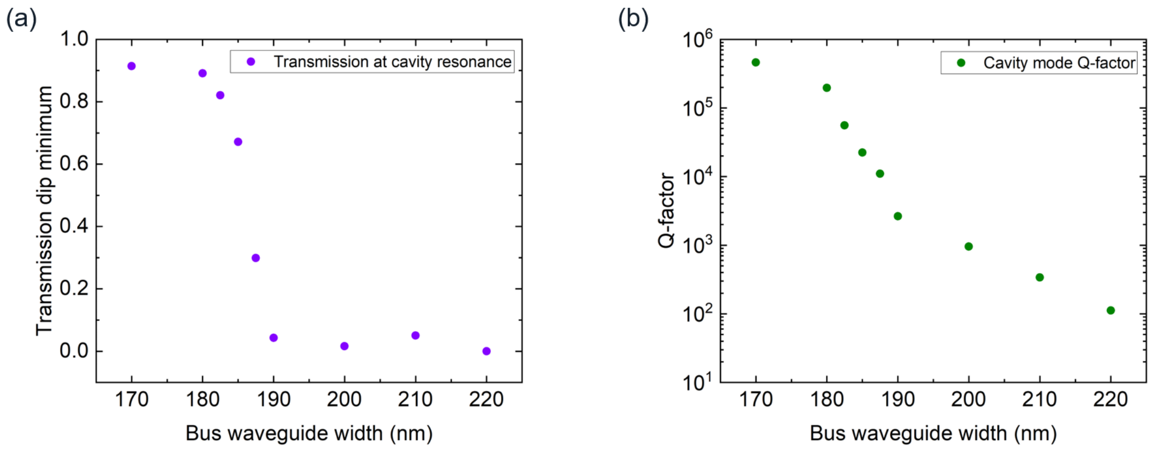

Figure S12: Simulations showing the relationship between the width of the bus waveguide and (a) end-to-end transmission through the waveguide (b) cavity mode Q-factor for a fixed coupling gap of $d_0 = 60$ nm.

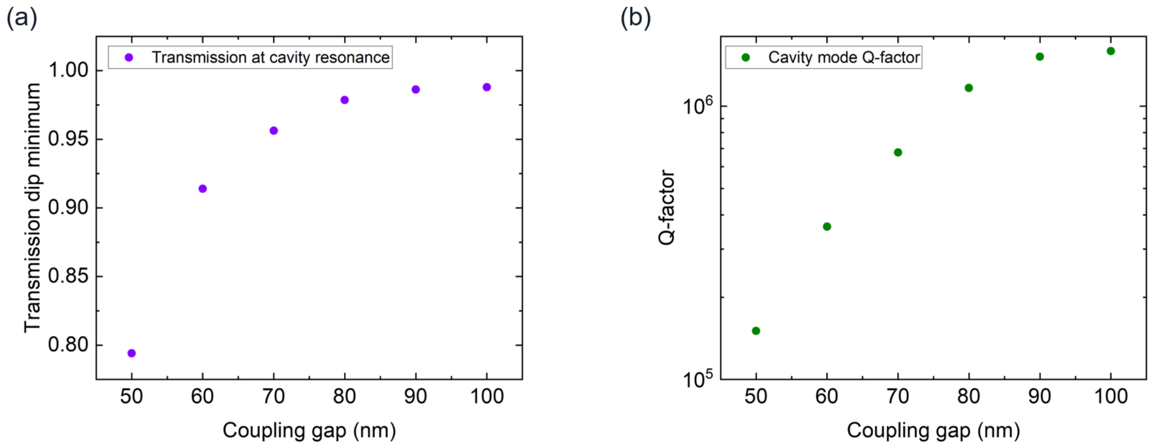

Figure S13: Simulations showing the relationship between the coupling gap and (a) end-to-end transmission through the waveguide (b) cavity mode Q-factor for a fixed bus waveguide width of $w_b = 170$ nm.

Figure S13 (a) and (b) show the relationship between the coupling gap and the transmission through the device, and cavity mode Q-factor for a fixed bus waveguide width, respectively. As the coupling gap is increased, the magnitude of the transmission dip decreases. This is due to the reduced spatial overlap between the cavity and waveguide modes reducing the coupling efficiency between the two modes. The Q-factor of the cavity mode also increases with increasing coupling gap due to this reduced coupling efficiency. For a fixed waveguide width of 170 nm, the influence of the coupling gap on the transmission dip and cavity Q-factor is totally diminished for $d_0 \geq 100$ nm.

Micro-PL

Figure S14 illustrates the optical setup used to obtain the results contained in the main text. The chip is placed in a bath cryostat at 4K. A CCD confocal camera is used to image the sample. The QD/cavity emission is collected in the far-field scattering directly over the cavity or OC, (refer to main text for each case) travelling through free space out of the device plane to couple optical pump power into the device or to propagate emission to a CCD spectrometer or Avalanche single-photon detector for analysis. All optical pumping was done using a above-band diode laser at 808 nm.

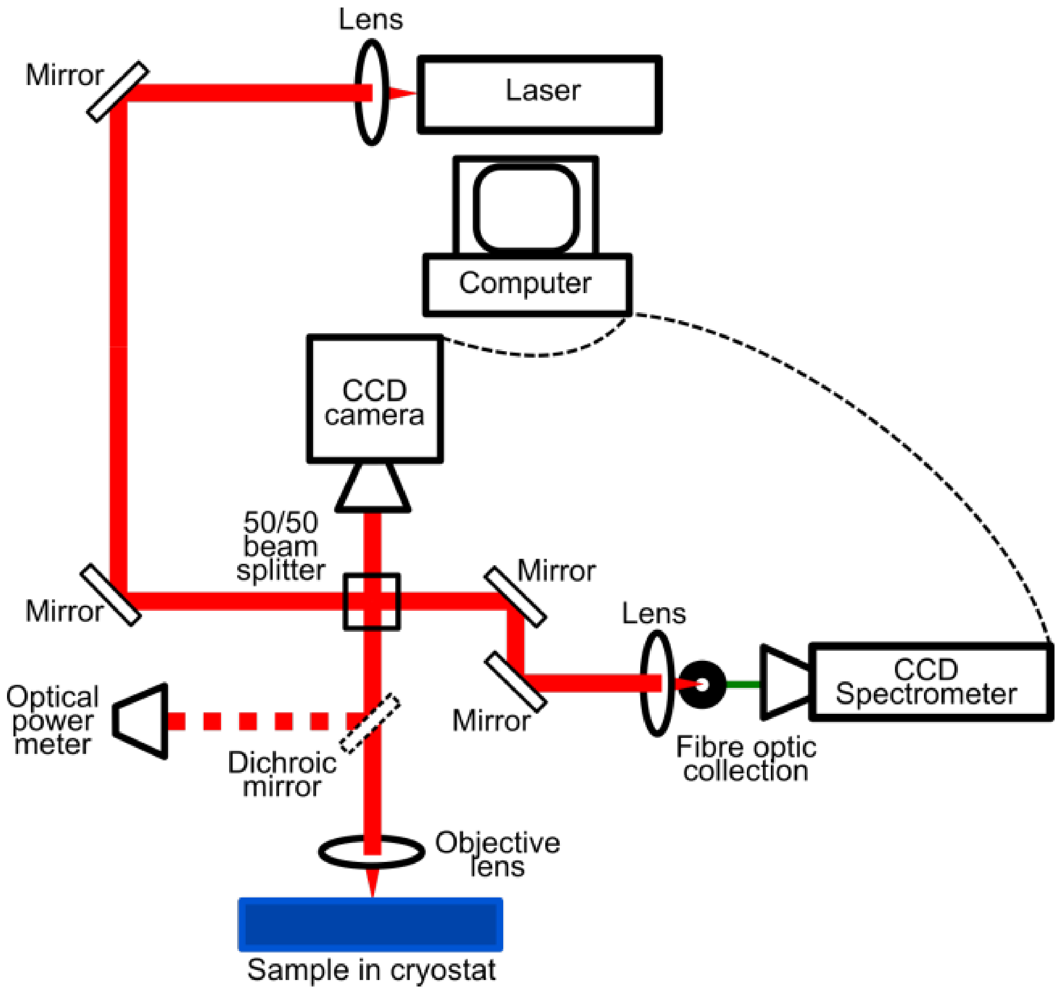

Figure S14: Diagram of Micro-PL setup

Cavity Coupled QD Cross Correlation Measurement

The model used to fit the second order cross correlation as a function of time delay between coincidence detection τ is given by:

$$g^2(\tau) = 1 - (1+a)e^{\frac{-|\tau|}{\tau_1}} + be^{\frac{-|\tau|}{\tau_2}}$$

Where :

- τ_1 is the anti-bunching characteristic time decay
- τ_2 is the bunching characteristic time decay
- a, b is the scaling of an imperfect system of anti-bunching and bunching respectively

Using a model which assuming b=1 we get the following fit:

**Parameter**	**Fitted Value**	**Error of Fit**
τ_1 (ns)	142.261	$\pm$ 17.350
τ_2 (ns)	439.907	$\pm$ 41.249
a	0.872	$\pm$ 0.135
$g^{(2)}(0)$	0.176	$\pm$ 0.142

Time Resolved Measurements

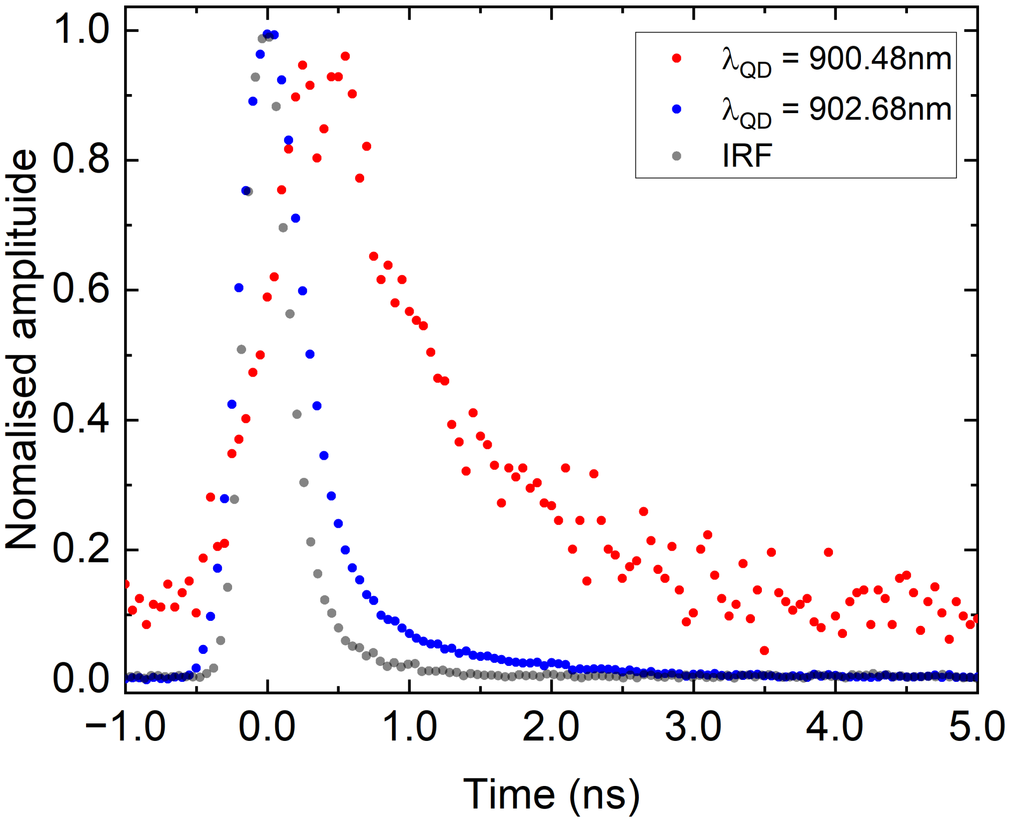

Figure S15: Fluorescence decay measurements of the QD studied in Figure 5 demonstrating the reduction in the radiative lifetime as the QD is tuned into resonance with he cavity mode. The measurement is resolution limited by the APD response time ($\sim$ 350 ps.)

Figure S15 shows the fluorescence decay measurements of the QD studied in Figure 5 at $\lambda_{QD} = 900.48$ nm and $\lambda_{QD} = 902.68$ nm, the slowest (red) and fastest (blue) decays measured, respectively. The instrument response function (grey) is also plotted demonstrating the resolution limitation of the measurement. We observe our fastest decay produces a response very similar to the instrument confirming the resolution limitation of the measurement.

References

(1) Brunswick, L. Nano-Photonic Structures for the Scale-up of III-V Semiconductor Based Quantum Optical Devices. Ph.D. thesis, University of Sheffield, 2022; https://etheses.whiterose.ac.uk/id/eprint/31776/.
